# Gender and neglected tropical disease front-line workers: Data from 16 countries

**DOI:** 10.1371/journal.pone.0224925

**Published:** 2019-12-19

**Authors:** Erica A. Shoemaker, Kelly Dale, Daniel A. Cohn, Maureen P. Kelly, Kathryn L. Zoerhoff, Wilfrid E. Batcho, Clarisse Bougouma, Georges B. Nko’Ayissi, Aboulaye Meite, Benjamin Marfo, André Goepogui, Marc-Aurele Telfort, Lita Renata Sianipar, Mahamadou Traore, Pradip Rimal, Djibo Aichatou Alfari, Chukwuma Anyaike, Fatou N. Badiane, Ibrahim Kargbo-Labour, Upendo J. Mwingira, Marcel S. Awoussi, Rachel D. Stelmach, Carly L. Smith, Jennifer Arney, Taroub Harb Faramand, Diana M. Stukel, Bolivar Pou, Lisa A. Rotondo, John D. Kraemer, Margaret C. Baker

**Affiliations:** 1 RTI International, Washington, DC, United States of America; 2 WI-HER, Vienna, Virginia, United States of America; 3 FHI 360, Washington, DC, United States of America; 4 National Communicable Disease Control Program, Ministry of Health, Cotonou, Republic of Benin; 5 National Neglected Tropical Disease Control Program, Disease Control Directorate, Ministry of Health, Ouagadougou, Burkina Faso; 6 Malaria and Neglected Tropical Diseases Sub-Department, Ministry of Public Health, Yaoundé, Republic of Cameroon; 7 National Control Program for Preventive Chemotherapy Neglected Tropical Diseases, Ministry of Health and Public Hygiene, Abidjan, Republic of Côte d'Ivoire; 8 National Neglected Tropical Diseases Program, Ghana Health Service, Accra, Republic of Ghana; 9 National Onchocerciasis and Blindness and Neglected Tropical Disease Control Program, National Prevention and Community Health Directorate, Ministry of Health, Conakry, Republic of Guinea; 10 National Malaria Control Program, Ministry of Public Health and Population, Port-au-Prince, Republic of Haiti; 11 Sub-Directorate of Filariasis & Helminthiasis Control, Directorate of Vector Borne Disease Control, Directorate General of Communicable Disease and Environmental Health, Ministry of Health, Jakarta, Republic of Indonesia; 12 National Schistosomiasis and Soil-Transmitted Helminths Control Program, Ministry of Health and Public Hygiene, Bamako, Mali; 13 Epidemiology and Disease Control Division, Department of Health Services, Ministry of Health, Kathmandu, Federal Democratic Republic of Nepal; 14 NTD Control Program, Directorate of the Protection of Public Health, Ministry of Public Health, Niamey, Republic of Niger; 15 Department of Public Health, Neglected Tropical Diseases Division, Federal Ministry of Health, Abuja, Federal Republic of Nigeria; 16 NTD Control Program, Disease Control Directorate, Ministry of Health and Social Work, Dakar, Senegal; 17 Neglected Tropical Diseases Programme, Disease Prevention and Control Directorate, Ministry of Health and Sanitation, Freetown, Republic of Sierra Leone; 18 Neglected Tropical Diseases Control Programme, Ministry of Health, Dar es Salaam, United Republic of Tanzania; 19 National Institute for Medical Research, Dar es Salaam, United Republic of Tanzania; 20 National Neglected Tropical Disease Control Program, General Directorate of Health, Ministry of Health and Social Welfare, Lomé, Republic of Togo; 21 Office of Infectious Diseases, US Agency for International Development, Washington, DC, United States of America; 22 Department of Health Systems Administration, Georgetown University, Washington, DC, United States of America; Centro de Pesquisa Gonçalo Moniz-FIOCRUZ/BA, BRAZIL

## Abstract

**Background:**

Delivery of preventive chemotherapy (PC) through mass drug administration (MDA) is used to control or eliminate five of the most common neglected tropical diseases (NTDs). The success of an MDA campaign relies on the ability of drug distributors and their supervisors—the NTD front-line workers—to reach populations at risk of NTDs. In the past, our understanding of the demographics of these workers has been limited, but with increased access to sex-disaggregated data, we begin to explore the implications of gender and sex for the success of NTD front-line workers.

**Methodology/Principal findings:**

We reviewed data collected by USAID-supported NTD projects from national NTD programs from fiscal years (FY) 2012–2017 to assess availability of sex-disaggregated data on the workforce. What we found was sex-disaggregated data on 2,984,908 trainees trained with financial support from the project. We then analyzed the percentage of males and females trained by job category, country, and fiscal year. During FY12, 59% of these data were disaggregated by sex, which increased to nearly 100% by FY15 and was sustained through FY17. In FY17, 43% of trainees were female, with just four countries reporting more females than males trained as drug distributors and three countries reporting more females than males trained as trainers/supervisors. Except for two countries, there were no clear trends over time in changes to the percent of females trained.

**Conclusions/Significance:**

There has been a rapid increase in availability of sex-disaggregated data, but little increase in recruitment of female workers in countries included in this study. Women continue to be under-represented in the NTD workforce, and while there are often valid reasons for this distribution, we need to test this norm and better understand gender dynamics within NTD programs to increase equity.

## Introduction

How societies construct gender—and the resultant gender roles, norms, expectations, and behaviors—has implications for the health workforce. It impacts, for example who is recruited for various positions, who has the opportunity for training and promotion, who is compensated and by how much, and what policies are in place to prevent workplace harassment. Even when opportunities for training or professional development are given to women, gendered responsibilities, norms, and access can limit their ability to take advantage of these opportunities.

Preventive chemotherapy (PC) is used to control or eliminate five of the most common neglected tropical diseases (NTDs)—lymphatic filariasis, onchocerciasis, schistosomiasis, trachoma, and a group of three soil-transmitted helminths [1] and is one of the world’s largest public health interventions. In 2018 alone, more than 1.7 billion treatments were delivered to more than 1 billion people [2]. Led by Ministries of Health (MOH), treatment is delivered through annual or biannual mass drug administration (MDA) by drug distributors—the NTD front-line workers. The drug distributors are trained, usually through a cascade training system, and overseen by supervisors. The profile of these drug distributors varies by country [3] from teachers and nurses to community volunteers who are selected and supported differently across countries [4].

Ensuring gender equity for these female and male drug distributors and supervisors is important. A recent report by the World Health Organization (WHO) states that addressing gender inequalities among front-line workers and ensuring decent work for men and women will not only improve gender equity, but also improve health, and spur greater economic growth [5].

The gender and sex of the drug distributors can also affect their ability to reach populations at risk of NTDs. While published evidence on these effects is limited, it reflects the importance of context and culture. In societies where women cannot discuss health with men, or women cannot let men into their homes, program success may require more female drug distributors; however, cultures where constructed masculinity discourages use of medications, male drug distributors may prove more effective in shifting the behaviors of their male peers [6,7].

It has been proposed that the sex of the drug distributor could also impact treatment coverage due to differences in working styles, but evidence of this effect is also limited and mixed. On the one hand, several studies show stronger female performance compared to male. A study in sub-Saharan Africa reported that during the distribution of ivermectin, surveyed community members frequently claimed that female drug distributors were more committed, persuasive, and patient than their male counterparts [4]. Similarly, Katabarwa *et al*. (2002)’s study in Uganda on the performance of female drug distributors found that increasing the number of female drug distributors potentially strengthened MDA programs [8]. In Tanzania, female drug distributors were reportedly more communicative with drug recipients and spoke in greater depth about trachoma prevention and facial cleanliness. However, these perceptions in themselves could also be a reflection of gender stereotypes. On the other hand, Chami *et al*. (2019) found that in the Mayuge District of Uganda, female volunteer drug distributors treated 12.0% fewer individuals. The authors could not determine decisively the reason for lower coverage rates amongst female drug distributors, but pointed to gendered roles and responsibilities outside of drug distribution work as a possible reason [9].

The literature on sex/gender and NTDS mainly focuses on biological differences in infection or social differences with respect to vulnerability to infection or access to services. Males and females have different reactions to and outcomes from NTDs due to biological susceptibility, as in the cases of female genital schistosomiasis (genital mucosal lesions) and hydrocele (enlarged scrotum caused by filarial worms). SCH further increases female’s risk for anemia and STH can lead to severe helminth-related anemia in pregnant females [10,11]. Gender norms and behaviors such as women’s responsibility for water collection, household sanitation, and caretaking in many communities makes them more vulnerable to NTD infections such as SCH and trachoma [12,13]. More traditional male occupational roles such as participating in agriculture and fishing expose men more to the parasites that cause SCH and STH [14,15]. When work and leisure activities keep men away from the home, their access to treatment during MDA may be reduced [16]. While consideration of the impact of gender on NTD infection and treatment efforts is growing, little research investigates the intersection of sex/gender and the NTD workforce.

Within the NTD literature there is a lack of sex-disaggregated data. Although Cohn *et al*. (2019) recently published a paper showing the rapid and significant increase in sex-disaggregated data for MDA in 16 countries supported by the United States Agency for International Development (USAID), which highlights both the feasibility of collecting this data and the power of donor recommendations [17], there still remain inconsistencies and gaps in disaggregated reporting standards across national programs. This paper seeks to address the gap in availability of published sex-disaggregated data by presenting an analysis of sex-disaggregated data of NTD front-line workers from 16 countries supported by USAID, using data collected on more than three million trainees. We pose and answer two questions. First, what are the trends in availability of sex-disaggregated data on NTD front-line workers? Second, what do the available data tell us about the male-to-female ratio of these workers?

## Methods

### Data source

This study uses data reported by national NTD programs in collaboration with USAID-supported NTD projects—ENVISION (managed by RTI International) and END in Africa (managed by FHI 360)—between USAID fiscal years (FY, October-September) 2012 and 2017 on training of front-line workers for MDAs. Training data is used as a proxy for the front-line MDA workforce, for which sex-disaggregated data is not available. These front-line workers for MDA are typically selected due to their role as teachers or community health workers (who are often selected by communities or local councils). Trainings typically occur just a week or two before the MDA itself, and national NTD program managers report that the majority of those trained go on to complete MDA.

Aggregate data on persons trained were routinely collected from project records and Ministry of Health (MOH) reports shared with local staff working on USAID-supported projects. Data were recorded in Microsoft Excel workbooks. These were sent to the ENVISION project headquarters at RTI International in the U.S. to be cleaned and subsequently reported to USAID. Data was securely stored in a password protected database that required users to sign a “user agreement” to ensure data security. Countries reported the number of persons trained by categorizing them into one of five predetermined groups: drug distributors, trainers, supervisors, monitoring and evaluation (M&E), or other. Trainers and supervisors were combined for purposes of our analyses because they typically occupy similar roles. We restricted our analyses to the drug distributors and trainers/supervisors categories because the remaining categories together comprised only a very small (6.3%) proportion of the total and were not trainees selected specifically for MDAs. Data were only included when MOH representatives gave written permission to use them. This resulted in 16 out of 25 countries being included in the analysis.

### Data cleaning

To check the validity of reported data, we conducted the following steps. (1) We verified that the sum of the number of males and females trained by job category was within ± 5% the total number trained. Three instances were re-evaluated based on this criterion; of them, we obtained corrected data for two and excluded one from the analysis. (2) We checked whether sex disaggregation was based on a formula. First, the percentage of females trained was evaluated to determine if it was between 49–51%, which could indicate that the total trained was simply divided evenly between males and females. Next, we checked reported sex-disaggregated data to make sure that they were not just a retrospective application of the fraction of the at-risk population expected to be each sex, but were instead sex-specific tallies. We identified no instances of sex disaggregation based on a formula.

Trends over time for each category, drug distributors and trainers/supervisors, were examined to inspect the data for potential errors in data entry. Two potential data-entry errors were identified and both were resolved after conferring with partner country offices.

### Measures and analyses

Principal variables of interest were sex (males and females as reported by programs), country, job category (as defined above: drug distributors and trainers/supervisors), and fiscal year.

First, we determined the availability of sex-disaggregated data by calculating the percentage of persons trained for which sex-disaggregated data were recorded by fiscal year. Then, we calculated the percentage of male and female trainees by country, job category, and fiscal year among those countries with disaggregated data. Our main analysis of sex-disaggregated data is on FY17 data, the most recent year and during which 100% of trainings reported disaggregated data. Data is not reported by disease, so we were not able to analyze sex-disaggregated data further disaggregated by each of the five NTDs. Because our data contain the universe of training from our target population in participating countries, we did not estimate confidence intervals. Analyses were conducted using SAS version 7.1, and figures were produced with R version 3.5.1.

This study is a secondary data analysis and analyzed only routinely-reported aggregate data from which no individual could be identified, so institutional review board approval was not required. This study is reported consistent with STROBE guidelines.

## Results

**Fig 1:**
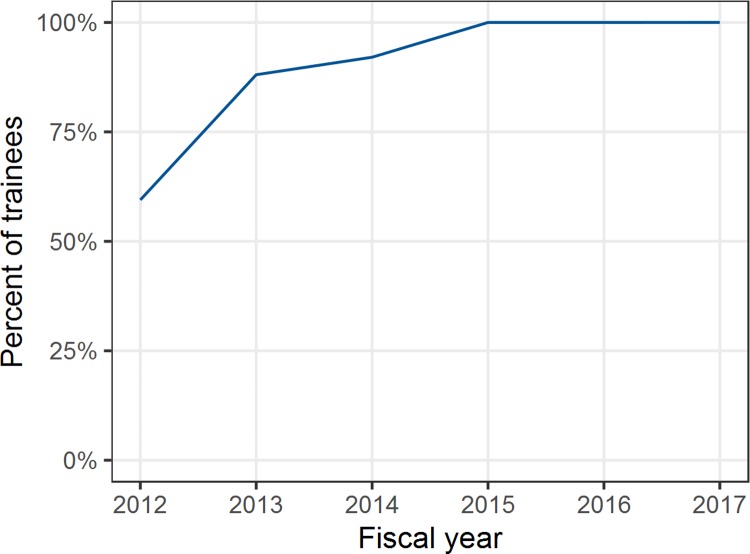
Percent of available sex-disaggregated data. This graph shows the percent of trainees that attended trainings where sex-disaggregated data was collected by fiscal year.

A total of 3,203,736 trainees were trained with USAID support from FY12-FY17. Of these, sex-disaggregated data were recorded for 2,984,908 trainees (93%). This percentage increased from 59% in FY12 to 88% in FY13 and then to 100% by FY15, which was sustained through FY17 (Fig 1).

**Fig 2:**
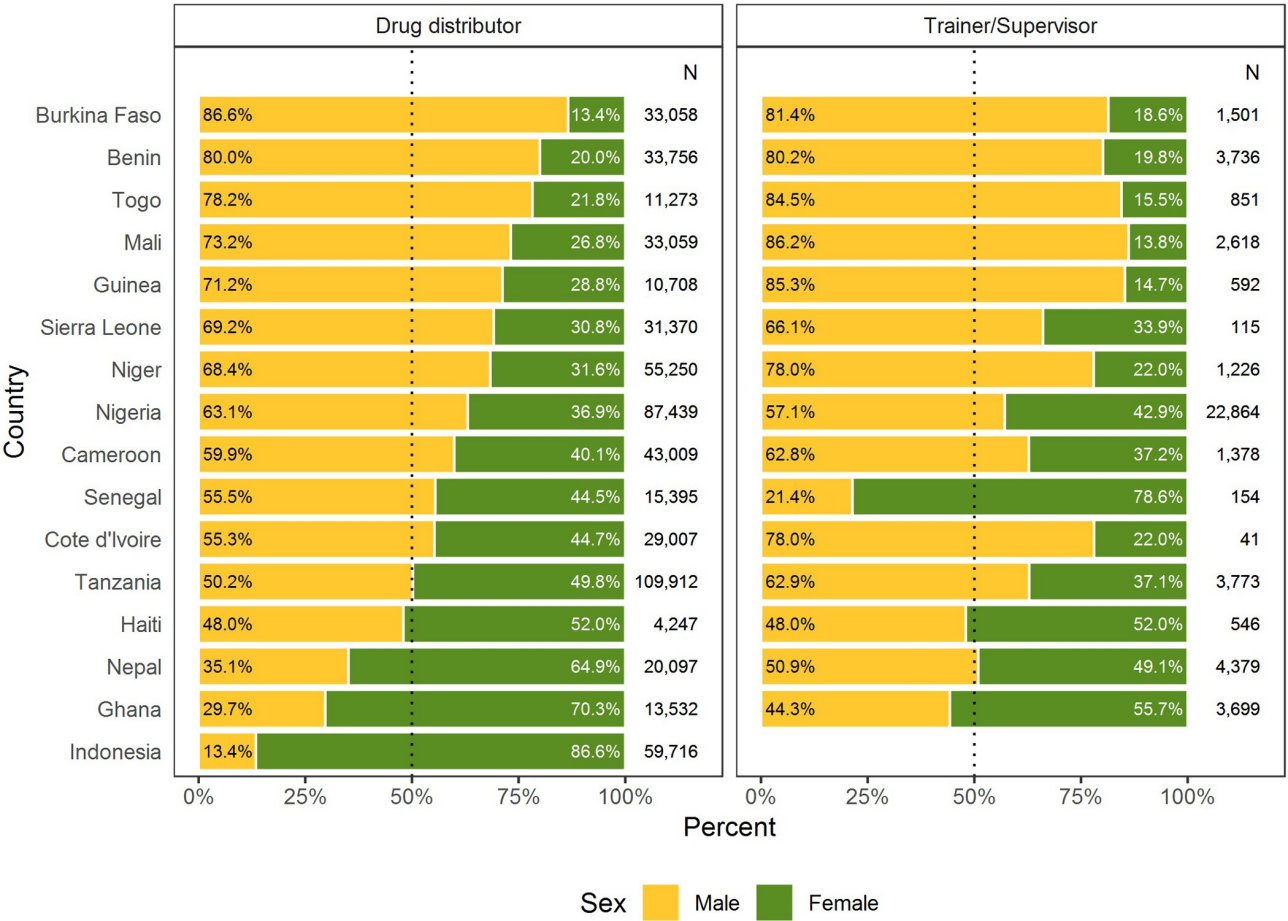
Percentage trained by country and job category in FY17. This graph represents the percentage of males and females trained in FY17 by country and job category. The total number of trainees are also listed by country and job category. No trainees participated in trainer/supervisor trainings for Indonesia in FY17.

Overall, in FY17, 43% of persons trained were female– 43% of drug distributors and 38% of supervisors/trainers. Across countries, the percentage of females trained as drug distributors ranged from 86.6% in Indonesia to 13.4% in Burkina Faso. Out of the 16 countries, 4 countries (Indonesia, Ghana, Nepal, and Haiti) trained more females than males as drug distributors. The percentage of females trained as trainers/supervisors ranged from 78.6% in Senegal to 13.8% in Mali, with 3 out of 15 countries (Senegal, Haiti, and Ghana) training more females than males (Fig 2).

**Fig 3:**
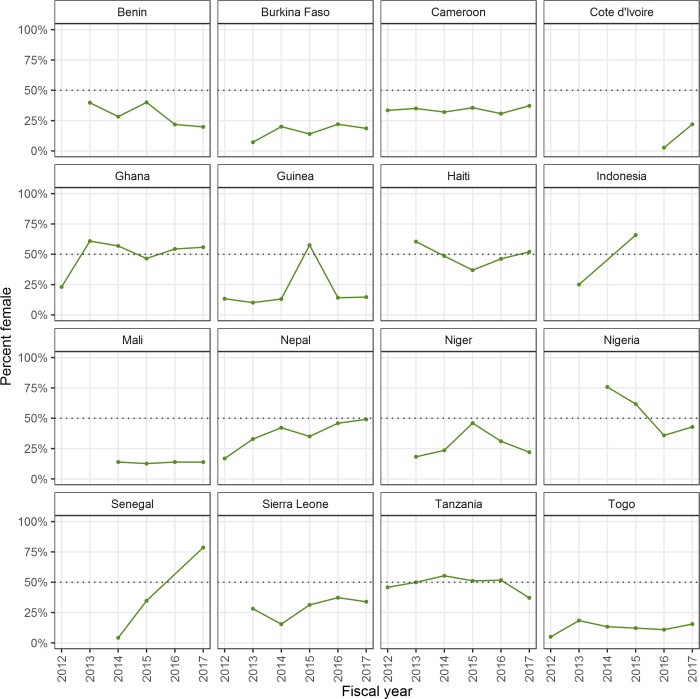
Percentage of females trained as trainers/Supervisors from FY12-FY17 by country. This collection of graphs shows the percentage of females trained as trainers/supervisors from FY12-FY17 by country. A dashed line is included at 50% for reference.

**Fig 4:**
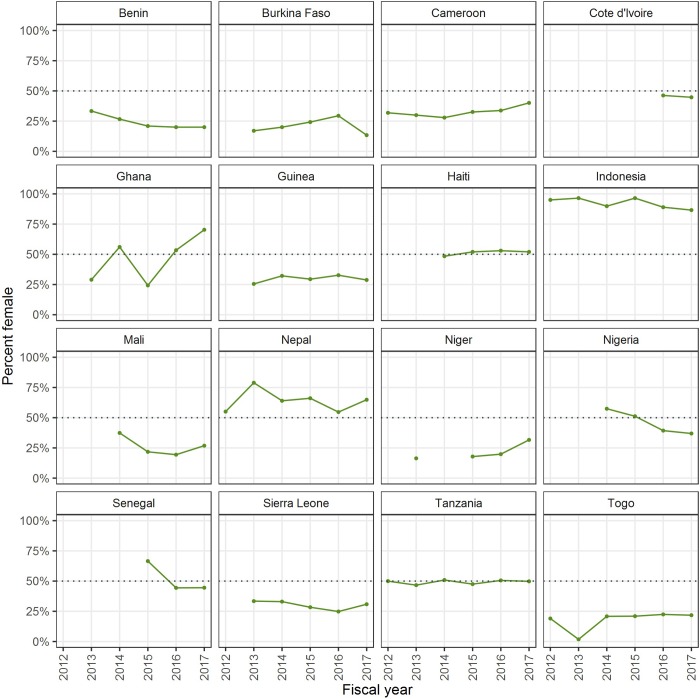
Percentage of females trained as drug distributors from FY12-FY17 by country. This collection of graphs shows the percentage of females trained as drug distributors from FY12-FY17 by country. A dashed line is included at 50% for reference.

When the percentage of females trained as drug distributors or trainers/supervisors are examined at a country level, there is little evidence of increases in female workforce, except for trainers/supervisors in Senegal and Nepal (Fig 3). Indonesia and Nepal consistently trained more females than males as drug distributors from FY12-FY17 (Fig 4).

## Discussion

The availability of sex-disaggregated data from trainings of NTD front-line workers increased rapidly from 59% FY12 to 88% in FY13 and reached 100% in FY15, which was sustained through FY17. This increase corresponds with implementation of the 2012 USAID policy requiring the collection and use of sex-disaggregated data in programming [18], including reporting sex-disaggregated data on persons treated [17]. Feedback from partner organizations and countries represented in this study showed that making this change was relatively straightforward in some countries, while other countries such as Senegal conducted supplementary sensitization with trainers and health staff to ensure that they understood the importance of this additional step and that data were collected properly. There were also national NTD programs that had begun reporting their training data disaggregated by sex before 2012. This was due to both national policies and cross-program learning in country. For example, Tanzania was required to report sex-disaggregated data for other health programs prior to 2012, which influenced how the NTD program collected their data. In Nepal prior to 2012, the government also required sex-disaggregation of data.

Despite increased availability of data on sex ratios, there is little evidence of increasing recruitment of female workers in most participating countries. The proportion of the NTD front-line workforce that was female ranged from those which reported a high proportion of female drug distributors (example: Indonesia, Ghana, and Nepal)—to those which reported mostly male drug distributors (example: Benin, Burkina Faso, and Togo). The percent of female trainers/supervisors was lower overall, except in Senegal.

Having sex-disaggregated data is an important foundation for being able to further understand and enhance gender equity in the health workforce. However, despite required collection of these data, it is not clear how much these data are being analyzed and used to inform policies and programmatic decisions. Several of the countries included in this study reported efforts made to use the sex-disaggregated data to influence change, with mixed results. For example, Ghana which has a majority-female workforce, has intentionally shared sex-disaggregated data at national and regional trainings. Burkina Faso, Togo, and Guinea, on the other hand, have tried to use the data to educate communities about the importance of female representation, with little change in the sex ratios of drug distributors in the years that followed, largely due to traditional customs and norms.

The composition of the NTD workforce may reflect broader systemic inequity in education and employment, as well as national policies and gender-related norms, roles, and responsibilities. In Sierra Leone, for example, women’s lower educational attainment and literacy levels limit their eligibility for drug distributor positions. Across multiple participating countries, NTD program managers also report that women may be less available due to their household and caregiving responsibilities.

The pre-existing occupation from which drug distributors are selected, and the ways in which they are selected, also impact male-to-female ratios. When NTD programs rely heavily on teachers and nurses to deliver the drugs or to supervise and train (e.g., Haiti and Senegal), the sex make-up reflects those professions, and NTD programs have limited influence over the composition of their drug distribution workforce. Other countries (e.g., Ghana, Mali, Tanzania, and Uganda) use community-based selection processes over which NTD programs may have limited influence due to local cultural and gender norms and biases.

Globally there are cultural and social differences that impact how people perceive and accept drug distributors. Therefore, unequal male-to-female ratios in front-line workers may also be designed to reflect local socio-cultural contexts. For example, Nigeria has strategically designed different regional strategies for drug distributors in response to religious and cultural norms: in the north, men cannot enter a house if a woman is alone, so having more female drug distributors is necessary. Female drug distributors may also not be recruited due to security concerns, especially where there is conflict or unrest.

It is also important to consider that recruiting women as drug distributors may also be putting them in harm’s way or be detrimental to their overall well-being. According to Isaacs (2018) and a recent report by WHO, community health workers are commonly subject to sexual harassment [5,19]. In Nepal, 42% of health workers (not NTD specific) have reported experiencing verbal and physical abuse, and community health workers in Kenya have reported rape and have experienced threats of violence by men when providing HIV testing to their wives [20,21]. Women often fear reporting cases of sexual harassment due to stigma or backlash, which results in limited data on the security of these health workers [5]. A study in Tanzania found that although community health workers—many of whom also serve as drug distributors—do their work out of intrinsic motivation, they and their families may struggle financially (due to low or no compensation), which places an unfair burden on these volunteer health workers and their family members [22]. Therefore, without concerted efforts to address these challenges, it is understandable why some women may not want to become a drug distributor.

Globally, women’s financial contribution to the health system amounts to approximately 5% of global gross domestic product (GDP), but about half of this contribution is unpaid [23]. Furthermore, the average gender pay-gap globally is around 16% [24]. Women and girls carry a disproportionately large burden of unpaid care work, which impacts the type and quality of work that is available to them and reinforces their disadvantaged economic status [5].

The path towards equity is not simple. Programs must balance empowering women in the NTD front-line workforce without putting them at increased risk of harm or exploiting them and their time. New guidance from the WHO encourages recruitment and selection procedures that maximize women’s participation and empowerment, stating that countries should aim for gender equity amongst volunteer health workers where culturally appropriate (ensuring acceptability of services by the target population). This is because employing female health workers can be empowering and offer a “critical opening for change towards achieving greater gender equity within communities [7].” Ensuring equity in the workforce also requires commitment beyond recruiting—there is a need for opportunities for advancement, equal renumeration, and policies to protect both men and women from harassment or maltreatment.

In most countries represented in this study, the NTD front-line workforce is made up primarily of males and has remained unchanged over the years. While there are sometimes valid reasons for this distribution, there is a need to better understand gender dynamics within NTD programs, in different contexts, and to test interventions to increase equity–in the workforce and of delivery to all persons at risk. We propose in Box 1 below several operations research questions that can be used to move this agenda forward.

**Box 1. Operational research questions.**

- What is the impact of the drug distribution role on women? When does it lead to increased empowerment, agency and opportunities, and when is it potentially exploitative?
- How do payment structures and other incentives affect the composition of the NTD workforce?
- Are men and women in the NTD workforce compensated equally for equal work? If not, why?
- Are more men selected as CDDs when the position is compensated (versus volunteer)?
- Does the type of MDA (school, household, or fixed-point) or the setting (urban, rural) affect the gender ratio of drug distributors?
- Do male and female drug distributors have similar retention rates? If not, what are the driving factors?
- How does the sex of the drug distributor affect program outcomes?
- How can we create policies and programs to achieve gender equity in the NTD workforce?
- How can programs better use the available sex-disaggregated data to make programmatic, policy, and human resource decisions?

### Limitations

There were several limitations that impacted our analyses. While we used a standardized data collection tool across all countries, there was no standard method for collecting the training data. The data collection tool also forced countries into selecting 1 of 5 pre-defined categories for each training, resulting a loss of some qualitative information. We cannot determine the number of unique individuals trained, as the same individual could be trained each year or counted in multiple trainings. Reports from our coauthors from countries included in the analysis indicated that the individuals trained typically represent the individuals that conducted the NTD work; however, individuals that participated in the training may not go on to participate in the MDA. The data were aggregated to the national level and therefore miss regional variation and only represents USAID-supported districts. Number of trainees trained are “as reported” by countries. Some trainings with the same topic took place in multiple locations or over multiple days and were reported as one training. In some countries, there are challenges in collecting sex-disaggregated data which could result in guessing the sex of the trainee based on their name, which inevitably leads to some biased or inaccurate results. However, these issues are expected to introduce a relatively small degree of error. Finally, the data used in this analysis is ultimately the property of each country’s respective MOH and each country was able to grant or deny permission based on their own unique circumstances. Countries that did not approve the use of their data were excluded from the analysis, so our results cannot be generalized beyond the countries included.

## Supporting information

S1 Table Detailed data. This workbook represents detailed information on the number of trainees that participated in USAID-supported trainings between fiscal years 2012–2017 by country, job category, and fiscal year.
(XLSX)

S1 File STROBE checklist. This checklist outlines the items that should be included in cross-sectional studies and where they can be found in this manuscript.
(DOC)

## References

[1] World Health Organization Preventive chemotherapy in human helminthiasis. Geneva, Switzerland; 2006.

[2] WHO. Update on the global status of implementation of preventive chemotherapy (PC). [Internet]. 2019. Available from: https://www.who.int/neglected_diseases/preventive_chemotherapy/PC_Update.pdf

[3] Baker M. Who are the NTD drug distributors? An analysis of platforms used for mass drug administration across multiple countries in symposuim 106 Community Providers for Neglected Tropical Disease Control: The “Building Blocks” for Program Success. ASTMH Conference 2016; 2016.

[4] Vouking MZ, Tamo VC, Tadenfok CN. Contribution and performance of female Community-Directed Distributors in the treatment of onchocerciasis with Ivermectin in Sub-Saharan Africa: a systematic review. Pan African Medical Journal [Internet]. 2015 [cited 2019 Feb 24];20. Available from: http://www.panafrican-med-journal.com/content/article/20/188/full/10.11604/pamj.2015.20.188.3337 PMC4577622 26430485

[5] World Health Organization. Delivered by women, led by men: A gender and equity analysis of the global health and social workforce. Geneva; 2019. (Human Resources for Health Observer Series No. 24).

[6] Jenson A, Gracewello C, Mkocha H, Roter D, Munoz B, West S. Gender and performance of community treatment assistants in Tanzania. Int J Qual Health Care. 2014;26(5):524–9. 10.1093/intqhc/mzu067 25022350 PMC4490229

[7] World Health Organization. WHO Guideline on Health Policy and System Support to Optimize Community Health Worker Programmes [Internet]. 2018. Available from: http://www.ncbi.nlm.nih.gov/books/NBK533329 30431747

[8] Katabarwa M, Habomugisha P, Agunyo S. Involvement and performance of women in community-directed treatment with ivermectin for onchocerciasis control in Rukungiri District, Uganda. Health Soc Care Community. 2002;10(5):382–93. 10.1046/j.1365-2524.2002.00378.x 12390224

[9] Chami GF, Kabatereine NB, Tukahebwa EM. Profiling the best-performing community medicine distributors for mass drug administration: a comprehensive, data-driven analysis of treatment for schistosomiasis, lymphatic filariasis, and soil-transmitted helminths in Uganda. BMC Medicine. 2019 3;17(69). 10.1186/s12916-019-1303-z PMC6437990 30917824

[10] Gouvras A. Neglected Tropical Diseases and Women; an International Women’s Day special [Internet]. BugBitten. 2017 [cited 2018 Oct 1]. Available from: https://blogs.biomedcentral.com/bugbitten/2017/03/10/ntds-deworming-women-international-womens-day-special/

[11] Brooker S, Hotez PJ, Bundy DAP. Hookworm-Related Anaemia among Pregnant Women: A Systematic Review. PLOS Neglected Tropical Diseases. 2008 9 17;2(9):e291 10.1371/journal.pntd.0000291 18820740 PMC2553481

[12] Seto EYW, Sousa-Figueiredo JC, Betson M, Byalero C, Kabatereine NB, Stothard JR. Patterns of intestinal schistosomiasis among mothers and young children from Lake Albert, Uganda: water contact and social networks inferred from wearable global positioning system dataloggers. 1. 2012 Nov 1;1–13. 10.4081/gh.2012.99 23242675

[13] Kiliminjaro Centre for Community Ophthalmology, The Elfenworks Foundation., The Carter Center. Women and Trachoma: Achieving Gender Equity in the Implementation of SAFE. Atlanta, GA; 2009.

[14] Yi-Xin H, Manderson L. The social and economic context and determinants of schistosomiasis japonica. Acta Tropica. 2005 11;96(2–3):223–31. 10.1016/j.actatropica.2005.07.015 16202596

[15] Guerra-Silveira F, Abad-Franch F. Sex Bias in Infectious Disease Epidemiology: Patterns and Processes. Nishiura H, editor. PLoS ONE. 2013 4 24;8(4):e62390 10.1371/journal.pone.0062390 23638062 PMC3634762

[16] Rilkoff H, Tukahebwa EM, Fleming FM, Leslie J, Cole DC. Exploring Gender Dimensions of Treatment Programmes for Neglected Tropical Diseases in Uganda. Gyapong M, editor. PLoS Negl Trop Dis. 2013 7 11;7(7):e2312 10.1371/journal.pntd.0002312 23875047 PMC3708858

[17] Cohn DA, Kelly MP, Bhandari K, Zoerhoff K, Batcho W, Drabo F, et al Gender equity in mass drug administration for neglected tropical diseases: data from 16 countries. International health. 2019; 10.1093/inthealth/ihz012 PMC6748770 30845318

[18] USAID. Gender Equality and Female Empowerment Policy [Internet]. Washington, DC: USAID; 2012 Mar. Available from: https://www.usaid.gov/sites/default/files/documents/1865/GenderEqualityPolicy_0.pdf

[19] Isaacs D. Sexual Harassment. Journal of paediatrics and child health. 2018;54(4):3410342. 10.1111/jpc.13877 29383784

[20] Prasad S, Bhusal K. Work place sexual harassment among female health workers in grass-root level health institutions in Nepal. Occuptational Medicine and Health Affairs. 2015;3(4).

[21] Steege R, Taegtmeyer M, McCollum R, Hawkins K, Ormel H, Kok M, et al How do gender relations affect the working lives of close to community health service providers? Empirical research, a review and conceptual framework. Social Science & Medicine. 209:1–13. 10.1016/j.socscimed.2018.05.002 29777956

[22] McCollum R, Gomez W, Theobald S, Taegtmeyer M. How equitable are community health worker programmes and which programme features influence equity of community health worker services? A systematic review. BMC Public Health. 2016;16(419). 10.1186/s12889-016-3043-8 PMC4875684 27207151

[23] Langer A, Meleis A, Knaul FM, Atun R, Aran M, Arreola-Ornelas H, et al Women and Health: the key for sustainable development. The Lancet. 2015 9;386(9999):1165–210. 10.1016/S0140-6736(15)60497-4 26051370

[24] International Labour Office. Global wage report 2018/19: what lies behind gender pay gaps. Geneva; 2018.

